# Complement activation in kidney transplantation

**DOI:** 10.1093/ndt/gfaf206

**Published:** 2025-10-03

**Authors:** Mehmet Kanbay, Lasin Ozbek, Mustafa Guldan, Zeynep Akcin, Can C Susal, Ahsen Morva Yilmaz, Sebahat Usta Akgul, Caner Susal

**Affiliations:** Division of Nephrology, Department of Medicine, Koç University School of Medicine, Istanbul, Turkey; Department of Medicine, Koç University School of Medicine, Istanbul, Turkey; Division of Nephrology, Department of Medicine, Koç University School of Medicine, Istanbul, Turkey; Transplant Immunology Research Center of Excellence TIREX, Koç University School of Medicine, Istanbul, Turkey; Department of Nephrology, Klinikum Stuttgart – Katharinenhospital, Stuttgart, Germany; Transplant Immunology Research Center of Excellence TIREX, Koç University School of Medicine, Istanbul, Turkey; TUBITAK Marmara Research Center, Climate & Life Science, Biotechnology Research Group, Kocaeli, Turkey; Transplant Immunology Research Center of Excellence TIREX, Koç University School of Medicine, Istanbul, Turkey; Transplant Immunology Research Center of Excellence TIREX, Koç University School of Medicine, Istanbul, Turkey

**Keywords:** graft rejection, complement system, ischemia-reperfusion injury, kidney transplantation, xenotransplantation

## Abstract

Long-term graft survival remains a challenge in kidney transplantation and, due to scarcity of available organs, solutions such as xenotransplantation have been revived, and testing of new agents, including complement-targeting biologicals with potential to improve transplant outcomes, has been enhanced. The complement system plays a critical role in the reaction of the host’s immune system to the transplanted foreign kidney. Activation of the complement system is strongly involved in ischemia/reperfusion-mediated injury and rejection of allografts as well as xenografts, and post-transplant recurrence rates are high in kidney diseases linked to complement system dysregulation. Therefore, the precise understanding of the complement activation cascade is important for the development of successful strategies that target complement before and after transplantation. This article compiles the current knowledge on the role of the complement system in kidney transplantation, examines recent developments in the clinical use of complement-targeting therapeutics and discusses the limitations of these approaches. While the current study results indicate that complement-targeting strategies are still in their early stages, promising findings related to the discovery of new agents encourage the redesign of existing therapeutic approaches and the development of more effective treatments.

## INTRODUCTION

**Figure 1:**
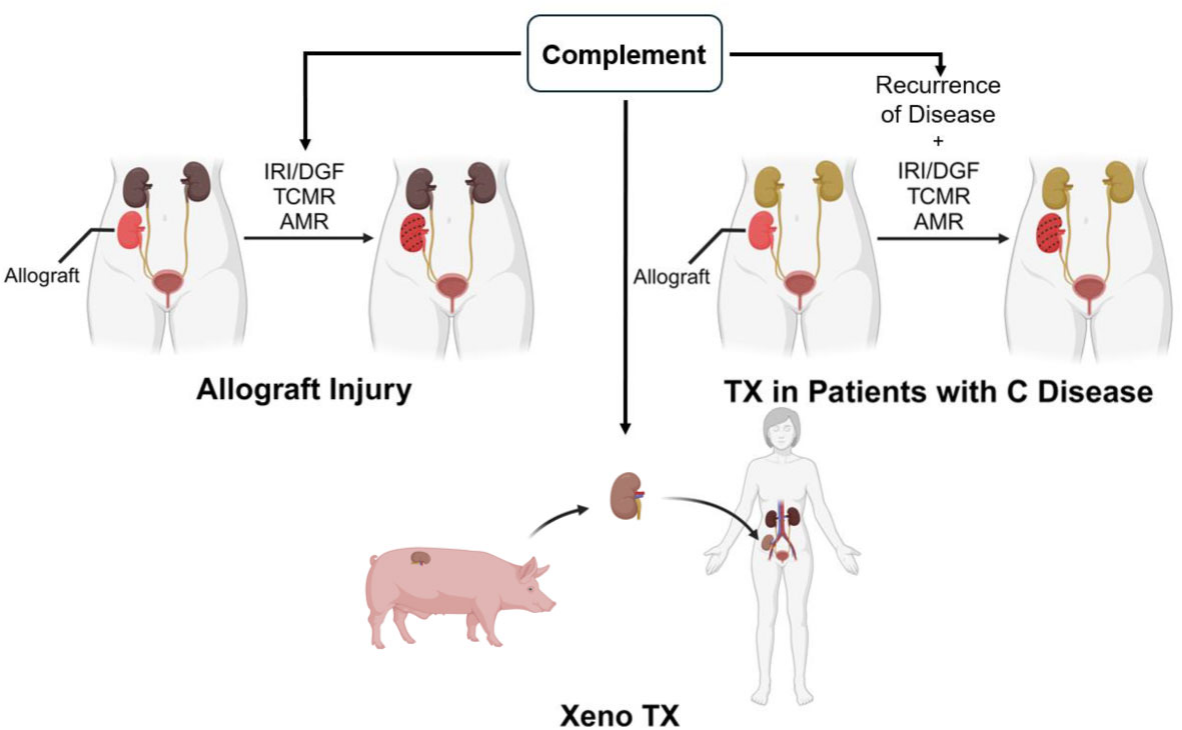
Role of complement in renal transplantation (TX). The complement system plays a critical role in processes such as IRI/DGF-mediated injury, and TCMR and AMR of renal allografts. IRI during the procurement and reperfusion of the graft and DGF can activate the complement system as a consequence of injury, exacerbate tissue damage within the graft, and adversely affect subsequent outcomes. Relapse of the primary disease is common in transplanted patients with complement (C)-mediated diseases such as aHUS, C3G and lupus nephritis. In pig-to-human xenotransplantation (Xeno TX), hyperacute rejection of the graft due to activation of the complement system by naturally occurring anti-pig gal antibodies represents a major hurdle.

Kidney transplantation is the most effective treatment strategy to improve survival and quality of life for patients with end-stage kidney disease. However, despite tremendous advances in transplant technology, the long-term outcomes of kidney transplants remain challenging [1]. The complement system plays a pivotal role in different forms of kidney allograft injury, contributing strongly to the impairment of long-term outcomes. In physiological conditions, activation of the complement system is involved in the clearance of microbes, immune complexes and damaged cells, whereas an aberrant activation of the complement system lurks at any stage of the transplantation process and, as depicted in Fig. 1, if activated strongly, leads to severe damage in the allograft through ischemic–reperfusion injury (IRI), delayed graft function (DGF), T cell– and antibody-mediated rejection (TCMR, AMR), recurrence of complement-related original disease and hyperacute rejection of the xenograft in the case of pig-to-human kidney transplants [1, 2]. In this overview article, we discuss the mechanisms through which the complement system contributes to allograft injury and evaluate the current state of therapeutic strategies that have been developed to control its activation and therefore have the potential to improve kidney transplant outcomes.

## THE COMPLEMENT SYSTEM

The complement system is an essential and dynamic component of innate immunity, serving as the body’s initial line of defense. With over 50 components, including soluble factors, cell surface receptors and regulatory proteins primarily synthesized in the liver, the complement system operates through a tightly controlled proteolytic hierarchy to facilitate enzymatic reactions that assist in opsonizing and lysing pathogens, removing damaged cells, clearing immune complexes, activating and modulating the immune response, and bridging innate and adaptive immune responses through complement-specific receptors on T cells [2].

Complement is activated through three distinct pathways, the classical pathway, the lectin pathway and the alternative pathway, each characterized by unique signaling mechanisms. These pathways are initiated by the binding of complement proteins either to an antibody that has bound antigen during a specific immune response or, in the absence of an antibody, directly to antigenic structures on pathogens and damaged cells. Regardless of the trigger, all three pathways converge in formation of homologous C3 convertase variants, cleavage of C3 by these convertases and generation of homologous forms of C5 convertase [2]. During the activation process, small “a” and larger “b” fragments are generated. The “a” fragments, e.g. C3a and C5a, are released into the circulation and act as anaphylatoxins and chemoattracts, whereas the “b” fragments, e.g. C4b, C2b, C3b, C5b and factor B (FBb), remain on the cell surface, act as opsonins and build convertases that cause cleavage of further components in the cascade [2].

### Pathways of complement activation

#### Classical pathway

The classical pathway is primarily activated by the binding of the first complement component, C1q and its associated proteases C1r and C1s to the Fc portion of IgG or IgM antibodies that have bound an antigen and formed an immune complex. The proteases C1r and C1s in the C1qrs complex initiate a cascade of proteolytic events that cleave C4 and C2 into their “a” and “b” fragments. The “b” fragments C4b and C2b form the C3 convertase enzyme C4bC2b on the cell surface, responsible for the cleavage of C3 into the anaphylatoxin C3a and the opsonin C3b [2].

#### Lectin pathway

The lectin pathway is triggered by the binding of pattern recognition molecules (PRMs) in the serum, such as mannose-binding lectin (MBL), ficolin and collectin-11, to fucosylated structures and abnormal carbohydrates, such as mannose, expressed on the surface of pathogens and damaged cells. Such binding activates MBL-associated serine proteases (MASPs), which, similar to C1r and C1s, cleave C4 and C2 into their “a” and “b” fragments that form the C3 convertase C4bC2b [2].

#### Alternative pathway

The alternative pathway, continuously active at a low level, amplifies complement activation by spontaneous hydrolysis of C3 to C3(H_2_O) that is able to bind and activate Factor B (FB). FB is cleaved by the serine protease Factor D (FD) into its “a” and “b” fragments, FBa and FBb. FBb form together with C3b bound on the surface of pathogens and damaged cells the alternative pathway C3 convertase C3bBb, which cleaves C3 into its small fragments C3a and C3b. Amplification occurs as C3bBb repeatedly cleaves C3, generating C3b which helps to form additional C3 convertases [2].

#### Convergence of three pathways leading to the activation phase

At the initiation phase’s conclusion, all pathways converge on C3’s cleavage into the fragments C3a and C3b. The deposition of C3b on surfaces promotes the formation of the C5 convertases C4b2b3b from the classical and lectin pathways and C3bBbC3b from the alternative pathway. These two convertases can cleave C5 into C5a and C5b, initiating the assembly of C5b, C6, C7, C8 and C9 to the membrane attack complex (MAC). MAC forms pores in target cell membranes, leading to osmotic cell lysis [2]. However, nucleated cells can resist lysis by expressing inhibitors (see below) that block the assembly of MAC. MAC can activate signaling cascades, such as the non-canonical nuclear factor-κB pathway, mediating processes like cell proliferation, adhesion molecule expression and release of proinflammatory cytokines interleukin (IL)-1β and IL-18 [2].

### Receptors and regulators of complement

A tight regulation of the complement cascade is critical to prevent excessive, uncontrolled activation. As shown in Table 1, complement fragments interact with different specific receptors (CR1, CR2, CR3, CR4, CRIg, C3aR, C5aR) on immune cells and exert various important functions [3]. In addition, membrane-bound complement inhibitors, such as decay-accelerating factor CD55 (DAF), membrane cofactor protein CD46 (MCP) and protectin (CD59), and soluble complement inhibitors, such as C4-binding protein (C4BP), Factor H (FH) and Factor I, play key roles in this regulation. Factor I, with the assistance of cofactors such as CD46, CR1, FH and C4BP, inactivates C3b and C4b, C1 inhibitor prevents early activation by inhibiting the protease activities of C1r and C1s and by binding of MBL to MASP-1 or MASP-2, and CD59 blocks formation of the MAC, thereby protecting host cells from damage [2, 3].

**Table 1:** Complement: receptors and their biological functions.

**Receptors**	**Ligands**	**Expressed on**	**Functions**
CR1	• C3b	• Erythrocytes	• Adhesion
	• C4b	• Phagocytes	• Immune modulation
		• B cells	• Inhibition of C3 and C5 convertases in classical and alternative pathways
			• Phagocytosis
CR2	• C3d	• B cells	• B-Lymphocyte activation
	• C3dg	• DCs	• B-Lymphocyte class switching
	• iC3b	• T cells	• Antigen retention on FDCs
CR3, CR4	• iC3b	Monocytes	• Adhesion
	• C3d	Macrophages	• Chemotaxis
		• Neutrophils	• Phagocytosis
		• Natural killer cells	
		• Activated T and B cells	
CRIg	• C3b	• Tissue resident macrophages	• Downregulation of IL-2 production
	• iC3b		• Inhibition of C3 and C5 convertases in classical and alternative pathways
			• Phagocytosis
			• T-cell proliferation
C3aR	• C3a	• Mast cells	• Chemotaxis
		• Basophils	• Inflammation
		• Phagocytes	
		• T cells	
C5aR	• C5a	• Neutrophils	• Chemotaxis
		• Macrophages	• Immune modulation
		• DCs	• Inflammation
		• T cells	• Phagocytosis

CR, complement receptor; C3aR, complement component 3a receptor; C5aR, complement component 5a receptor; FDCs, follicular dendritic cells; iC3b, inactivated C3b.

## COMPLEMENT AND KIDNEY TRANSPLANTATION

**Figure 2:**
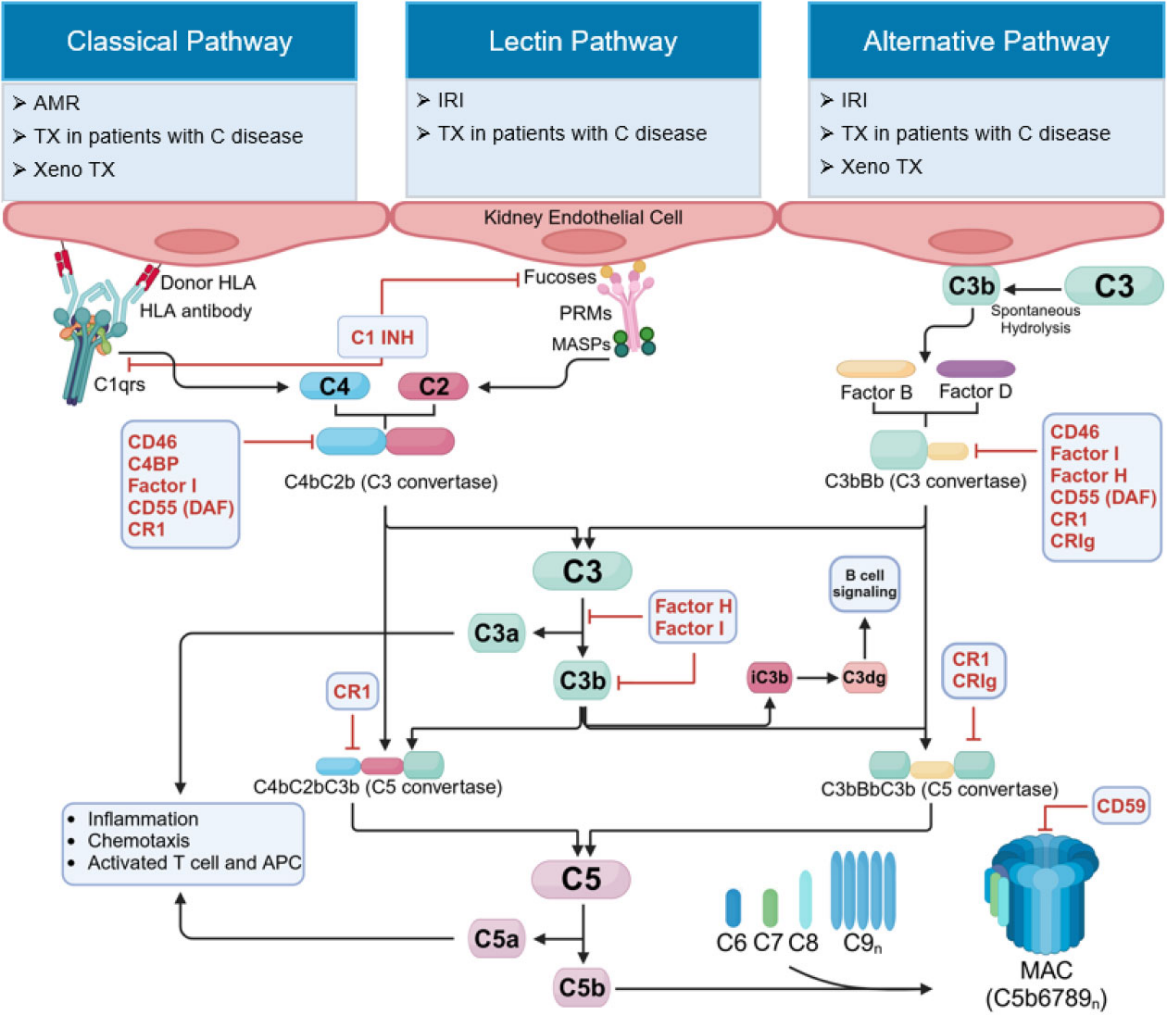
Pathways, components and regulators of the complement system in organ transplantation (TX). Complement (C) system can be activated through multiple pathways in response to different stimuli. Antigen-antibody complexes on foreign surfaces initiate the classical pathway, while polysaccharides, lipopolysaccharides and IgA antibodies activate the alternative pathway. The lectin pathway is activated as a result of the interaction of PRMs with specific carbohydrate structures, such as fucose, leading to the activation of MASPs. All three complement activation pathways converge with the cleavage of C3 into C3b and C3a by the C3 convertases C4b2b (classical and lectin pathways) and C3bBp (alternative pathway), which starts the terminal pathway tract and ultimately ends in formation of the MAC through the assembly of C5b-C9. AMR after transplantation, including C-related disease relapse, proceeds through the classical pathway. In xenotransplantation (Xeno TX), the classical as well as the alternative pathways can be activated. Additionally, C3b, by converting to C3dg, promotes the activation of B cells, enhancing their proliferation and antibody production. IRI triggers the lectin pathway by releasing endogenous DAMPs from acutely damaged tissue. Additionally, release of anaphylatoxins C3a and C5a after cleavage of C3 and C5 promotes recruitment of inflammatory cells, secretion of pro-inflammatory cytokines/chemokines and reactive oxygen species, thereby enhancing tissue necrosis and apoptosis. Key regulators of C activation include C1 inhibitor (C1-INH), FH, Factor I, complement receptor (CR)1, CRIg, CD46, C4b-binding protein (C4BP), CD55 (DAF) and CD59. C1-INH inhibits activation of C1r and C1s, preventing the cleavage of C4 and C2. Moreover, C1-INH can inhibit the binding of MBL to MASP-1 or MASP-2. Factor I and FH, assisted by CD55, CR1, CRIg and CD46, regulate C3 convertase activity in the alternative pathway. Factor I and FH, in addition, participate in inactivation of C3b. CR1 and CRIg inhibit activation of C5 convertase. CD59 inhibits assembly of C9 with C5b678, thereby preventing formation of MAC. APC, antigen-presenting cell.

The transplanted kidney, as a highly stress-sensitive organ, interacts closely with the immune system. While the liver is the main site of complement production, renal parenchymal cells (glomerular epithelial, endothelial, mesangial and tubular cells) are also able to synthesize their own complement proteins [1, 2]. In particular the tubular regions are dependent on local complement production, whereas glomeruli, in addition, also have access to the complement factors of liver origin [2]. Moreover, the intracellular active complement system, termed the “complosome,” plays an important role in maintaining endothelial and epithelial cell integrity and function in the kidney [4, 5]. The complement system plays a critical role not only in diseases such as antibody-mediated glomerular diseases and thrombotic microangiopathies (TMAs) but also in IRI and rejection of organ transplants. Hereby, activated complement fragments, particularly C3a, C5a and MAC, can induce cell death and fibrosis in the kidney [1, 6]. Figure 2 illustrates the pathways, components and regulators of complement that are involved in different forms of complement-mediated allograft damage.

The donor’s primary disease, e.g. immunoglobulin A nephropathy (IgAN) or atypical hemolytic uremic syndrome (aHUS, a subtype of TMA in new terminology), comorbidities like diabetes, source of the graft (deceased or living donor), graft preservation techniques and duration, immunosuppressive treatment regimens, and health status of the recipient significantly affect the inflammatory microenvironment and determine the extent of complement-mediated damage [2, 6].

### Role of complement in ischemia–reperfusion injury

**Figure 3:**
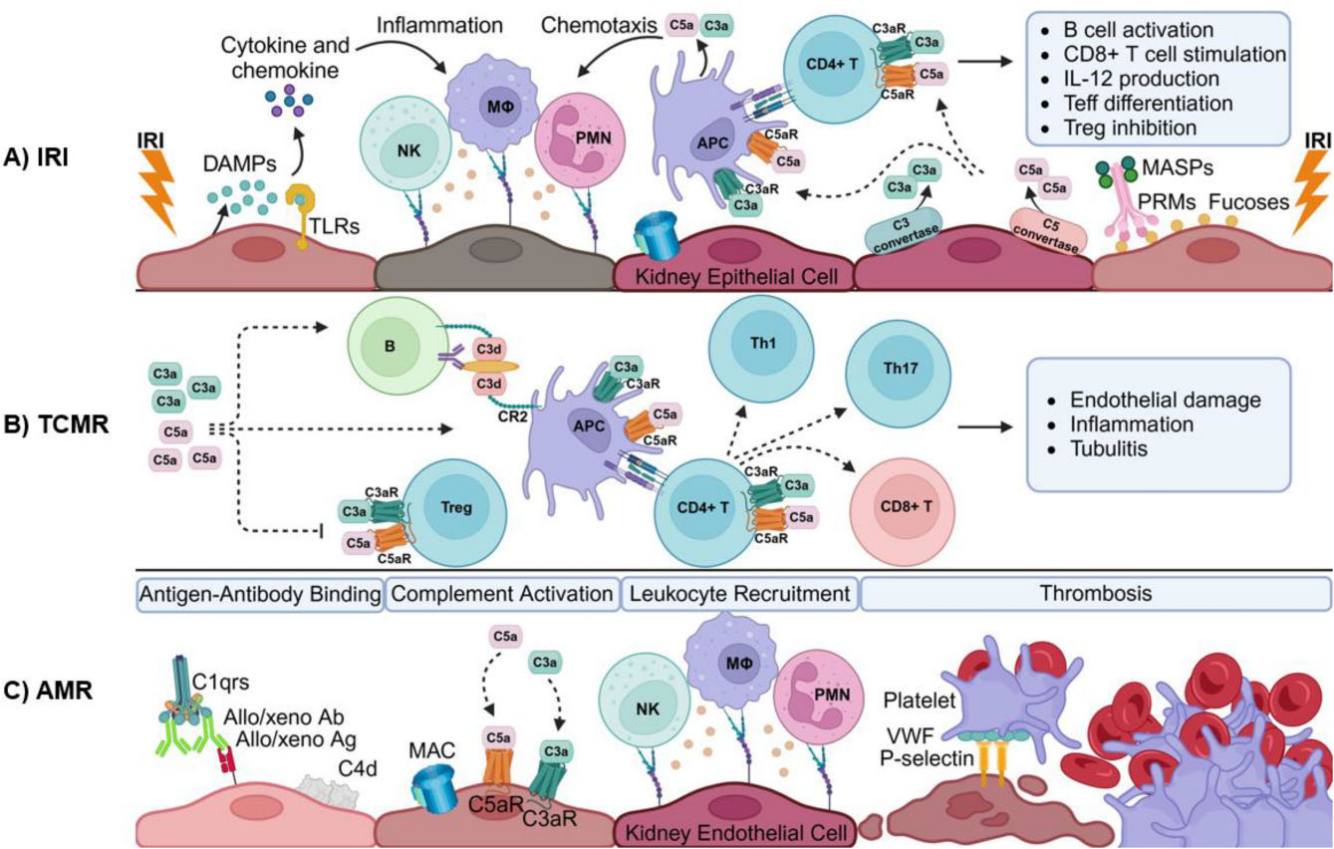
Role of complement in renal allograft injury. Key immunological events related activation of the complement system during allograft injury are described. (**A**) IRI triggers complement activation via the lectin pathway by exposing stress-induced ligands, including fucosylated structures, on damaged tissues, recognized by PRMs such as MBL, ficolin and collectin, along with their associated serine proteases (MASPs). The resulting complex binds to the altered glycocalyx of damaged cells and contributes to complement activation. Additionally, DAMPs activate the innate immune system through Toll-like receptors (TLRs), promoting the production of pro-inflammatory cytokines and chemokines. This leads to the activation of macrophages (Mø), polymorphonuclear leukocytes (PMNs), and natural killer (NK) cells, as well as the stimulation of antigen-presenting cells (APCs) to initiate adaptive immune responses. Complement activation results in the generation of anaphylatoxins C3a and C5a, which further enhance inflammation and recruit immune cells. These mechanisms exacerbate immune activation and injury, contributing to graft dysfunction. Endothelial and leukocyte adhesion receptors depicted in the figure, including intercellular adhesion molecule 1 (ICAM-1), vascular cell adhesion molecule 1 (VCAM-1) and selectins, mediate leukocyte recruitment and adhesion during IRI. Additionally, the alternative pathway also plays a role in IRI; this aspect has not been depicted in the figure. (**B**) In TCMR, C3a and C5a trigger inflammation, activate immune cells and support T-cell differentiation. The binding of C3a and C5a to C3aR and C5aR on APCs enhances antigen presentation, while their binding to these receptors on T cells regulates the inflammatory response. C3d facilitates B-cell activation by promoting the retention of antigens on APCs. Additionally, the binding of C3d-opsonized antigens to complement receptor (CR) 2 on B cells lowers the activation threshold, thereby promoting B-cell activation and antibody production. In summary, anaphylatoxins contribute to the activation of CD8^+^ T and B cells, proliferation of T effector (Teff), Th17 and Th1 cell populations, and inhibition of Treg cells. Endothelial damage, inflammation and tubulitis develop during this process. (**C**) AMR begins when DSAs bind to the graft’s endothelial cells, triggering the classical complement activation pathway. C5b-9 (MAC) forms pores in the cell membrane, initiating endothelial cell lysis. Low levels of MAC and C5a trigger the exocytosis of Weibel-Palade bodies, increasing the release of von Willebrand factor (VWF), which further intensifies inflammation. P-selectin interacts with complement activation, enhancing the expression of complement products and directly activating complement. Soluble complement fragments C3a and C5a, as chemotactic factors, promote the migration of inflammatory cells to the graft site. These cells cause microcirculatory inflammation such as peritubular capillaritis and glomerulitis. The binding of C3a and C5a to endothelial receptors triggers the expression of adhesion molecules, cytokines and chemokines, leading to leukocyte accumulation, platelet adhesion and increased vascular permeability. An important product of complement activation, C4d, covalently binds to the endothelial surface and serves as a biomarker of AMR.

IRI, as one of the major non-immunological adverse events in kidney transplantation, induces inflammation that can result in cellular damage and DGF [7]. A key component of the IRI-initiated inflammatory response is activation of the complement system, which amplifies tissue injury, both locally and systemically, as a consequence of oxidative stress during reperfusion (Fig. 3A) [5].

Experimental data from allogeneic murine transplantation models have confirmed that IRI impairs graft function not only via circulating recipient-derived complement components but also through local complement production by donor tubular epithelial cells [8]. During IRI, complement synthesis is markedly upregulated by renal endothelial cells, tubular epithelial cells, and infiltrating immune cells. Supporting this, syngeneic rat transplantation studies (i.e. in the absence of alloimmune responses) have demonstrated a burst in C3 mRNA expression within glomeruli as a consequence of ischemic injury [9].

Under physiological conditions, the endothelial glycocalyx layer and membrane-bound complement regulatory proteins, such as CD46 (MCP), CD55 and CD59, as well as fluid-phase regulators, like FH, C1 esterase inhibitor (C1-INH) and C4BP, control and limit complement activation. However, during ischemia, glycocalyx degradation via metalloproteinases and heparanase leads to damage and loss of these regulators, rendering endothelial cells vulnerable to unregulated complement activation [5]. Furthermore, a shift toward anaerobic metabolism and subsequent acidosis promotes formation of the alternative pathway C3 convertases, facilitating complement activation through the alternative route [2, 3]. In addition, breakdown of the glycocalyx and release of damage-associated molecular patterns (DAMPs) from injured endothelial and epithelial cells result in recognition by natural IgM antibodies and pattern recognition molecules such as MBL and collectin-11, initiating activation via the classical and lectin pathways. Notably, collectin-11 plays a key role in local lectin pathway activation by binding to l-fucose structures upregulated on ischemic tubular epithelial cells [2, 10].

The central role of the alternative pathway in IRI has been robustly demonstrated in preclinical models. Mice deficient in C3 or FB exhibit significantly reduced inflammation and tissue damage following renal ischemia, highlighting the pathogenic importance of this pathway [11]. Similarly, therapeutic blockade of FB using monoclonal antibodies was shown to suppress complement activation and protect against necrotic and apoptotic tubular injury in murine models [11].

The lectin pathway also plays a critical role in IRI. In mice lacking MBL, MASP-2 or collectin-11, various models of renal, cardiac and cerebral ischemia showed preserved tissue integrity, reduced inflammatory infiltration and decreased cell death [5, 10, 12]. Particularly, activation of MASP-2 by collectin-11, independently of C4, has been emphasized. In mice deficient in both MASP-2 and C4, only MASP-2 deficiency conferred protection against tissue damage, indicating that lectin pathway activation can proceed via a C4-independent mechanism. Consistent with these findings, pharmacological inhibition of MASP-2 with EVO24L significantly reduced complement activation, inflammation and renal tissue injury [13].

The role of the classical pathway in IRI remains controversial. Previous studies have reported that C4-deficient mice were not protected from renal IRI, and that C1q-deficiency in myocardial IRI even led to larger infarct sizes compared with wild-type controls, suggesting a limited contribution of the classical pathway [14, 15]. However, other experimental work has demonstrated that hypoxia can activate both the classical and alternative pathways, and that classical pathway activation contributes to ischemic injury in skeletal muscle and intestine [16, 17]. This apparent inconsistency may be explained by organ-specific anatomical or physiological differences. In the kidney, complement activation seems to predominantly affect parenchymal cells, whereas in the heart and other organs, the endothelium may be the main target. In contrast, the lectin pathway consistently emerges as a major contributor to ischemic injury in cardiac and gastrointestinal transplant models [14].

In conclusion, the ischemia–reperfusion process activates the complement system primarily through the alternative and lectin pathways, resulting in acute tissue injury. Disruption of local complement regulation, acidic microenvironments, DAMP-mediated signaling and glycocalyx degradation collectively exacerbate this response. The dysregulated activation of complement not only delays early graft function but may also increase the risk of subsequent rejection. Thus, targeted complement inhibition, particularly via FB, FD, MASP-2, C3 or C5 blockade, represents a promising therapeutic strategy to mitigate IRI and improve both short-term graft function and long-term transplant outcomes.

### Role of complement in T cell–mediated rejection

TCMR is characterized by the excessive activation of T cells against graft antigens, placing T cells at the center of the adaptive immune response. The complement system functions not only as a component of innate immunity but also as a critical regulator of adaptive immune responses [1].

As illustrated in Fig. 3B, the interaction of complement fragments C3a and C5a with their respective receptors, C3aR and C5aR, on antigen-presenting cells and T cells can modulate T cell proliferation, activation and differentiation, thereby shaping the intensity and direction of the adaptive immune response [18]. Such signaling creates an inflammatory microenvironment within the graft, promoting tissue injury [18]. The effects of C3a and C5a are not limited to cell activation; C5a–C5aR signaling can also enhance expression of the anti-apoptotic molecule Bcl-2 and suppress Fas expression, thereby preventing apoptotic cell death. This prolongs T-cell survival and contributes to the persistence of the inflammatory response [19].

Locally synthesized C3 by dendritic cells (DCs) plays a central role in shaping alloreactive T-cell responses. Donor DC-derived C3 enhances the surface expression of co-stimulatory molecules such as major histocompatibility complex class II and B7.2 and promotes T helper (Th) 1-polarized responses via IL-12 secretion. In contrast, C3 deficiency impairs the antigen-presenting capacity of DCs and Th1 polarization while upregulating IL-4, IL-10, and Foxp3 expression, thus favoring Th2 and regulatory T cell (Treg) responses [20]. Furthermore, Toll-like receptor activation triggers autocrine C3aR/C5aR signaling in DCs, which amplifies proinflammatory T cell responses and contributes to graft tissue injury [21].

Inhibition of the complement system may provide significant advantages in the induction of immune tolerance. Specifically, blockade of C3aR and C5aR promotes the conversion of CD4⁺ T cells into Tregs, enhances Foxp3 expression and stabilizes Treg phenotype, preventing their conversion into effector T cells [22].

### Role of complement in antibody-mediated rejection

In ABO incompatibility, pre-existing IgM-type antibodies to ABO blood group antigens are potent activators of the complement system. In HLA-incompatible kidney transplantation, binding of alloantibodies developed against mismatched donor human leukocyte antigen (HLA) class I and II molecules (DSA) can trigger inflammatory processes through both complement-dependent as well as complement-independent mechanisms. Inflammation in the graft promotes upregulation of adhesion molecules on the endothelium and production of chemokines and cytokines, thereby exacerbating vascular inflammation [23]. Through various pathways, from antigen trapping to acting as a molecular adjuvant, the complement system acts as a crucial orchestrator in the perplexing mechanisms governing humoral immunity and AMR (Fig. 3C). For quite a while, the cardinal role of the complement system in AMR has been discounted despite its dual function in inducing endothelial damage and stimulating B cells for antibody production. Feucht *et al*. were the first to show the close relationship between the complement system activation and AMR by demonstrating the abundant deposition of tissue-bound complement split products C4d and C3d in biopsy samples of patients with AMR [24]. Activation of the classical pathway continues with MAC formation, leading to complement-dependent cytotoxicity (CDC), responsible for endothelial cell lysis by promoting an inflammatory cascade and vasculature disruption [1]. MAC also supports the humoral response by mounting an alloreactive type 1 T-cell response with the release of interferon-gamma (IFN-γ) cytokines. IFN-γ increases HLA class I and II expression on endothelial cells, leading to enhanced antibody-mediated complement activation on the cell surface [5]. However, although the capillary deposition of C4d is an important diagnostic marker for AMR, C4d-negative AMR cases also exist.

In a notable study, C3 complement inhibitor Cp40 was shown to significantly prolong allograft survival in sensitized nonhuman primates, effectively preventing AMR and promoting graft survival despite high levels of DSA [25]. In the same study, C1q- and C3d-fixing *de novo* DSA were found to be useful markers for predicting AMR and graft outcomes [25].

Nonetheless, the precise involvement of the complement pathways in AMR remains insufficiently understood. Feucht *et al*. demonstrated that C5b-9 deposition was absent in C4d-positive AMR biopsies and that C5b-9 expression was only mild across all groups, suggesting that the contribution of the terminal complement cascade to AMR pathogenesis may have been overestimated [26]. Moreover, no significant difference in peritubular and glomerular C5b-9 deposition was observed between acute AMR and non-rejection biopsies [26]. On the other hand, C5b-9 positivity was reported in some AMR case studies treated with the C5 inhibitor eculizumab, indicating that terminal complement inhibition may be effective in a subset of patients, particularly those with early-onset rejection and high HLA antibody reactivity [1]. Therefore, whether eculizumab can be broadly applied in AMR remains debatable. More likely, C5b-9 plays a pathogenic role only in selected AMR cases, and such patients should be accurately identified prior to treatment [1].

In summary, understanding the mechanisms of complement activation reveals the causes of graft injury in transplantation and highlights the complement system as an important therapeutic target.

### Transplantation of patients with complement-mediated disease

The regulation of the complement system can be disrupted by conditions such as genetic dysregulation or the release of autoantibodies. Diseases such as complement-mediated (CM)-TMA, complement 3 glomerulopathy (C3G), IgAN and systemic lupus erythematosus are associated with abnormal complement activation [2]. Genetic variants and the effects of immune complexes highlight the role of the complement system in the pathogenesis of these diseases [2].

Systemic lupus erythematosus is a typical autoimmune disease in which autoantibodies activate all three complement pathways, leading to systemic hyperinflammation [2]. In rare kidney diseases like aHUS and C3G, mutations in FH or FH-related (FHR) genes or antibody-mediated activation of the alternative pathway can lead to glomerular damage. aHUS, mediated in 30% of cases by FH mutations, is characterized by a high post-transplant recurrence rate and poor prognosis [1]. During kidney transplantation, high immunosuppressive drug levels or infections can activate the complement system and trigger recurrence of aHUS or development of *de novo* TMA, which occurs in 0.8%–14% of kidney transplant cases [1].

Rare kidney diseases, especially those with pathogenic variants in complement genes, carry a high recurrence risk in transplanted kidneys [27]. Post-transplant glomerulonephritis is the third most common cause of allograft loss [27]. Studies have shown that complement-mediated membranoproliferative glomerulonephritis (MPGN) cases exhibit a higher tendency for early recurrence compared with immune complex-mediated MPGN [27]. Additionally, patients with C3G/Ig-MPGN experience high recurrence rates after transplantation [27].

These findings highlight the critical role of complement system regulation in the pathogenesis of complement-mediated diseases and indicate that targeting the complement system has the potential to reduce the recurrence risk, improving survival of kidney grafts [1].

### Role of complement in xenotransplantation

Given their biological and physiological similarities to humans, pigs have become the primary source for xenotransplantation [28, 29]. Nevertheless, xenogeneic antigens such as α-Gal [galactosyl-α(1, 3)-galactose], Neu5Gc (N-glycolylneuraminic acid) and Sd(a) (Sid antigen), which are absent in humans but present on pig organs, trigger potent antibody- and complement-mediated responses, leading to a high risk of hyperacute rejection that conventional immunosuppression can only transiently suppress [30]. To overcome this barrier, donor pigs have been genetically engineered to express human complement regulatory proteins (CD46, CD55, CD59), providing an effective strategy to mitigate complement-mediated graft injury [31]. Pig kidneys modified with the *GGTA1KO*/*hCD55* gene combination showed survival beyond 100 days in rhesus monkeys; in monkeys with low non-gal antibody titers, selective depletion of CD4^+^ Th or CD8^+^ cytotoxic T lymphocytes achieved the longest kidney xenotransplant survival of 499 days [32, 33].

The first xenotransplantation experiments conducted in brain-dead humans demonstrated that GGTA1 KO–modified pig kidneys did not undergo hyperacute rejection but experienced functional loss in the long term. Additional genetic modifications, including complement inhibitors (hCD55, hCD46), anticoagulant genes (hTBM, hEPCR) and immunomodulatory molecules (hCD47, hHO1), may further reduce immune responses [34]. Recent pioneering clinical xenotransplants of genetically modified pig hearts, kidneys and livers into humans demonstrated that such grafts can overcome early immunological rejection and maintain short-term function, although long-term efficacy and safety remain uncertain [35–37].

In conclusion, advances in xenotransplantation and complement system modulation provide a promising solution to the shortage of transplantable organs. Through genetic modifications and complement-targeting therapies, the immune response to donor organs can be controlled, paving the way for successful clinical applications.

## CLINICAL PERSPECTIVE ON COMPLEMENT ACTIVATION: IMPLICATIONS FOR PROGNOSIS AND PATIENT AND GRAFT OUTCOMES

The extent to which complement system activation impacts graft and patient outcomes has been investigated to explore therapeutic strategies aimed at preventing post-transplant complications. In a prospective cohort study in which C3a, C4a and C5a levels were assessed in 190 lung transplant recipients, changes in C4a and C5a levels were associated with primary graft dysfunction (PGD), while C3a and C5a levels were linked to mortality, independent of PGD. Notably, an increase in C5a levels between 6- and 24-h post-transplant was associated with both PGD and mortality [38].

A study found that complement deposition was more pronounced in DGF, AMR and TCMR biopsies compared with control groups, with differential abundance and localization of complement factors observed [1]. Early complement activation correlated with increased serum creatinine levels and morphological changes in biopsies and was suggested to have a predictive role for inferior graft outcomes. Patients with circulating complement-activating DSA were reported to be at higher risk of graft loss and rejection [1]. Additionally, mutations in complement genes appeared to increase the risk of graft loss and recurrence in aHUS patients [1]. Altogether, these findings show a strong correlation between complement activation and inferior graft outcomes, and suggest that targeting complement with therapeutics has great potential to improve the outcomes in kidney transplantation.

## THERAPEUTIC APPROACHES TARGETING THE COMPLEMENT SYSTEM IN TRANSPLANTATION

Currently, numerous clinical and preclinical studies are underway on complement-targeting therapies [e.g. eculizumab, C1 Esterase inhibitor (C1-INH), BIVV009]. These drugs are being investigated to reduce risks in organ transplantation, prevent rejection, treat AMR, and mitigate DGF and IRI (Supplementary data, Table S1) [1].

### Eculizumab

Eculizumab is a monoclonal antibody that inhibits the complement protein C5, preventing the formation of the terminal complement complex and MAC formation [39]. It is Food and Drug Administration–approved for treating paroxysmal nocturnal hemoglobinuria and aHUS. Beyond its approved uses, eculizumab also shows promise for preventing AMR and C3G in renal transplantation [40, 41], with ongoing trials exploring its potential in IRI and DGF [42, 43]. In kidney transplant recipients treated with eculizumab, an uncontrolled retrospective study reported graft failure rates of 20% for CM-TMA and 23.1% for AMR [44]. Eculizumab also showed some positive outcomes in highly sensitized individuals and those with HLA/ABO incompatibilities, reducing AMR incidence [1, 45, 46], yet requires more evidence. A phase 2 study demonstrated a graft survival rate of 83.4% at 36 months in recipients with preformed DSA. Eculizumab also served as a prophylactic therapy in living-donor recipients, preventing acute AMR [45]. However, there was no significant difference in the primary endpoint, and reductions in AMR rates were observed only in a sensitivity analysis or upon reassessment of the pathology data. Indeed, its impact on long-term chronic AMR risk remains uncertain.

A pilot study showed that eculizumab might improve estimated glomerular filtration rate (eGFR) changes in recipients with newly developed DSA, though statistical significance was lacking [47]. While generally safe and effective, eculizumab use has limitations such as infections and graft failure. In pediatric transplant recipients, eculizumab showed better early graft function but increased graft loss due to infections [42]. A trial depicting the eculizumab’s effect on DGF has shown no significant differences in DGF incidence, eGFR or patient survival [43]. Eculizumab has been effective in preventing aHUS recurrence, particularly in patients with mutations in complement regulatory proteins like FH [48], and has improved prognosis in complement-mediated hemolytic uremic syndrome (cHUS) patients [48].

In conclusion, while eculizumab improves graft function in AMR and aHUS recurrence, its effectiveness in IRI remains uncertain. The high costs, infection risks and need for meningococcal vaccination pose challenges [49]. The impact on T cell–mediated injuries needs further investigation, and it may be beneficial for patients with high DSA reactivity or signs of complement cascade activation [1].

### C1 Esterase inhibitor

C1-INH is a glycosylated serine protease inhibitor used to treat hereditary angioedema by inhibiting the classical complement pathway, preventing kallikrein and factor XII activation, and reducing bradykinin production [50]. It also limits proinflammation and coagulation by clearing C3 and C4 fragments [50].

In transplantation, C1-INH has shown promise in managing AMR and improving post-desensitization outcomes. A phase 2b study of plasma-derived C1-INH for acute AMR following kidney transplantation found improved renal function and prevention of transplant glomerulopathy, though graft survival was unaffected [51]. Another study with high-dose intravenous immunoglobulin and C1-INH Berinert showed improved eGFR and immunological benefits in refractory AMR patients [52]. A phase 1/2 study demonstrated that C1-INH reduced AMR incidence, decreased C1q^+^ HLA antibodies, and resulted in fewer adverse events [53]. These findings suggest C1-INH’s potential in preventing AMR, warranting further investigation.

C1-INH has also shown effectiveness in reducing IRI and DGF risk by inhibiting complement activation, decreasing inflammation and tubulointerstitial damage [50]. In a placebo-controlled trial with kidney transplant recipients at risk for IRI and DGF, C1-INH reduced dialysis requirements, improved renal function and lowered graft loss rates without increasing adverse events [54].

Overall, while C1-INH shows potential in transplantation, further larger and longer-term studies are needed to confirm its efficacy.

### Other therapeutic agents

While eculizumab and C1-INH are the primary agents targeting complement activation in renal transplantation, other compounds are also being investigated for alternative mechanisms.

Mirococept (APT070) is a membrane-targeted C3 convertase inhibitor that can be administered *ex vivo* to the donor kidney immediately prior to transplantation. The (Efficacy of Mirococept (APT070) in Preventing Ischemia/Reperfusion Injury in Kidney Allografts) EMPIRIKAL trial was designed as a double-blind randomized controlled study to evaluate the efficacy of Mirococept in reducing DGF in cadaveric kidney transplantation [55]. Initial low doses (5–25 mg) did not show a significant effect, prompting a dose-finding study in a porcine kidney model, which identified an optimal dose of 80 mg (equivalent to 120 mg in humans) without inducing additional histologic injury or systemic adverse effects [55]. Building on these findings, the ongoing EMPIRIKAL-2 phase 2a multicenter trial aims to determine the safest and most effective dose of Mirococept (60, 120 or 180 mg) to prevent DGF in deceased-donor kidney transplantation, with dialysis requirement in the first post-transplant week as the primary endpoint [56]. These studies collectively provide a robust preclinical and clinical framework for the potential use of *ex vivo* Mirococept to mitigate IRI in renal transplantation.

Pegcetacoplan, a targeted C3 and C3b inhibitor that blocks the activity of both C3 and C5 convertases across the classical, lectin and alternative pathways, has shown potential to mitigate complement-mediated glomerular injury. Clinical studies indicate that pegcetacoplan provides therapeutic benefit in C3G and demonstrates a favorable safety profile across multiple glomerular diseases [57]. In particular, the phase 2 (Novel Blockade of C3 for Evaluation in C3 Glomerulopathy) NOBLE trial established its efficacy, safety and tolerability in patients with post-transplant recurrent C3G and primary IC-MPGN, supporting further development of this agent as a rational therapeutic option in complement-driven nephropathies [58].

Antibodies that inhibit MASP-2, such as narsoplimab, initially showed encouraging signals in early-phase studies in IgAN [59]. However, the phase 3 (A Randomized, Double-blind, Placebo-controlled Study to Evaluate the Efficacy and Safety of Narsoplimab in IgA Nephropathy) ARTEMIS-IgAN trial did not meet its primary endpoint of proteinuria reduction at Week 36 compared with placebo [60], thereby limiting the enthusiasm for this approach in IgAN. In contrast, inhibition of the alternative pathway has shown more robust results. FB inhibitors, such as iptacopan, were first suggested by early studies to be beneficial in IgAN [61]. More recently, a phase 3 trial demonstrated that iptacopan significantly reduced proteinuria and was well tolerated, providing strong evidence for its efficacy and safety in adults with biopsy-proven IgAN [62]. These findings establish alternative pathway inhibition as one of the most promising complement-targeted strategies in kidney diseases, while lectin pathway inhibition with MASP-2 inhibitors has not yet translated into clear clinical benefit. Alternative pathway inhibitors, particularly FB inhibitors, are being investigated not only in IgAN but also in C3G and recurrent C3G after kidney transplantation [63]. Iptacopan, by targeting complement activation in C3G, has emerged as the first disease-specific treatment option. The pivotal phase 3 (A Phase 3 Study to Evaluate the Efficacy and Safety of Iptacopan (LNP023) in Adult Patients With C3 Glomerulopathy) APPEAR-C3G trial enrolled adult patients (≥18 years) with biopsy-proven native C3G (native cohort) or recurrent post-transplant C3G (transplant cohort) and an eGFR ≥30 mL/min/1.73 m^2^. Iptacopan was well tolerated and demonstrated dose-dependent, sustained inhibition of the alternative pathway in both cohorts. At Week 12, treatment with iptacopan led to a statistically significant reduction in urine protein-to-creatinine ratio in patients with native C3G and a significant reduction in histologic C3 deposition scores in patients with recurrent C3G after transplantation [64]. These results provided the basis for the regulatory approval of iptacopan in 2025 for adults with C3G [65, 66].

In addition, monoclonal antibodies targeting complement component C2, such as ARGX-117, are currently under investigation in a phase 2, placebo-controlled trial in deceased donor kidney transplant recipients at risk for DGF. This study aims to evaluate the safety, efficacy and tolerability of ARGX-117, reflecting the potential of complement-targeted therapies to improve early allograft function and reduce ischemia–reperfusion related injury [67].

Ongoing trials and future therapies with optimal drug dosing and larger patient cohorts will clarify the efficacy of these agents and potentially lead to new treatments.

## CURRENT TRIALS AND FUTURE DIRECTIONS

In addition to eculizumab and C1-INH, other agents targeting the complement system may show potential in the treatment of post-transplant organ injury. DAF, a complement regulator expressed in podocytes, has been an important target in research aimed at preventing hyperacute rejection [68]. Therapeutic strategies that inhibit DAF cleavage show promise in preventing focal segmental glomerulosclerosis by blocking C3a/C3aR signaling [69].

The lectin pathway was identified as a mechanism leading to reperfusion injury, and MASP-2 has emerged as a therapeutic target to inhibit this pathway. Additionally, antibodies that inhibit MASP-2, such as narsoplimab, have shown some positive results in the treatment of IgAN [59].

The agents mentioned are only examples for numerous other complement modulators that range from regulatory proteins such as soluble MCP and soluble CD59, Serping 1 and FH, to specific antibodies for complement cascade products [70].

Although eculizumab and C1-INH are the most studied therapies in solid organ transplantation, many trials have been prematurely terminated due to inefficacy or enrollment difficulties. Publication bias and other inhibitors that have not yet provided sufficient evidence should also be considered. The promising upcoming results hold potential not only for transplantation therapy but also for treating autoimmune diseases, where the complement system plays a role. While the focus of this paper is on renal transplant recipients, it is important to note that complement therapeutics have also been extensively studied and reported in the management of thrombotic microangiopathies following hematopoietic stem cell transplantation. For example, ravulizumab, although not currently under investigation in solid organ transplant recipients, has been studied in several clinical trials for its effects on TMA in hematopoietic stem cell transplant, another important focus in complement activation (NCT04557735, NCT04784455) [71]. Additionally, ongoing research on complement inhibitors is exploring potential applications in treating diseases beyond organ transplantation (Table 2).

**Table 2:** Complement: inhibitors tested in trials beyond organ transplantation.

**Compound**	**Target**	**Class**	**Disease**
AMY-101 (Cp40)	C3	Peptide	C-mediated diseases
APL-1 (POT-4)	C3	Peptide	AMD, geographic atrophy
Tesidolumab (LFG316)	C5	Antibody	AMD, HSCT-TMA, NIU, PNH
Cemdisiran (ALN-CC5)	C5	RNAi	aHUS, GN, IgAN, PNH
Zimura (ARC 1905)	C5	Aptamer	AMD, IPCV, Stargardt’s macular dystrophy
Cemdisiran (ALXN5)	C5	Antibody	aHUS, gMG, GN, IgAN, IIM, PNH, healthy controls
Coversin	C5	Protein	AKC, PNH
Zilucoplan (RA101495)	C5	Peptide	gMG, PNH
Crovalimab	C5	Antibody	aHUS, PNH
SOBI002	C5	Antibody	Healthy controls
Olendalizumab (ALXN-1007)	C5a	Antibody	aGVHD, aPL nephropathy, healthy controls
Vilobelimab (IFX-1)	C5a	Antibody	GPA, HS, SCC of skin, Severe COVID-19 pneumonia, SIRS
Avacopan (CCX168)	C5aR	Small Molecule	aHUS, ANCA-associated vasculitis, C3G, HI, IgAN, healthy controls
TP10 (sCR1)	C3 and C5 convertases	Protein	Aortic valve insufficiency, C3G, DDD
TT30	CR2–FH	Protein	PNH

AMD, age-related macular degeneration; HSCT-TMA, hematopoietic stem cell transplantassociated thrombotic microangiopathy; NIU, noninfectious uveitis; PNH, paroxysmal nocturnal hemoglobinuria; GN, glomerulonephritis; aHUS, atypical hemolytic uremic syndrome; gMG, generalized myasthenia gravis; IIM, idiopathic inflammatory myopathy; AKC, allergic keratoconjunctivitis; GPA, granulomatosis with polyangiitis; HS, hidradenitis suppurativa; SCC, squamous cell carcinoma; SIRS, systemic inflammatory response syndrome; aGVHD, acute graft-versus-host disease; aPL, antiphospholipid; HI, hemolytic illness; DDD, dense deposit disease.

## CONCLUSIONS

Kidney transplantation is the therapy of choice for patients with end-stage kidney disease. However, the post-transplantation period is often complicated by serious events, particularly due to the excessive activation of the complement system. The complement system plays a central role in graft rejection, IRI and other immune-mediated damage, making it a significant challenge in kidney transplantation [1]. A deeper understanding of the complement system has paved the way for the development of innovative therapeutic strategies to mitigate these complications.

Specific inhibitors targeting the complement system hold great potential for graft protection and organ rejection prevention [1]. Notably, complement inhibitors were shown to significantly reduce recurrence rates of transplant-related diseases caused by complement-associated conditions and improve overall transplant success rates [1]. Despite these advancements, critical questions remain regarding the appropriate timing for initiating and discontinuing complement inhibitor therapy as well as which patient populations will benefit most from these treatments.

Ongoing developments in complement system modulation place this field at a significant frontier in transplant medicine. These therapeutic strategies show potential not only for organ transplantation but also for treating autoimmune and inflammatory diseases [2]. Recent advance in xenotransplantation holds promise for alleviating the issue of organ shortage [33].

In conclusion, complement system modulation stands out as one of the most promising research areas in transplant medicine. Further investigation in this field will lead to the emergence of more effective treatment protocols and herald a revolutionary era in organ transplantation. These advancements will not only improve graft outcomes but also make the entire transplantation process safer and more effective, ultimately contributing to the evolution of the field and expanding treatment options.

## Supplementary Material

gfaf206_Supplemental_File

## Data Availability

No new data generated in this manuscript.
