# Supplementary material for: Complement activation in kidney transplantation
Source: Nephrol Dial Transplant. 2025 Oct 3;41(5):816–27. doi: 10.1093/ndt/gfaf206 (PMC13161900; doi:10.1093/ndt/gfaf206)
Supplement: gfaf206_Supplemental_File [file gfaf206_Supplemental_File.docx]

**Supplementary Table 1.** Previously Reported and Ongoing Clinical Trials in Organ Transplant Recipients with Complement-Inhibiting Agents*

| **Intervention** | **Target** | **Phase** | **Status** | **Trial Number** | **Indication** | **Population** | **N** | **Outcome** | **Limitations**** |
| --- | --- | --- | --- | --- | --- | --- | --- | --- | --- |
| **Allograft Injury Due to AMR** | | | | | | | | |  |
| C1-INH Berinert | C1r/s; MASPs | 1/2 | Completed | NCT01134510 (1) | Prevention of AMR | Highly HLA-sensitized kidney TX recipients | 20 | Berinert group vs placebo: serious AE 1 vs 2; DGF 1 vs 4; AMR 0 vs 3; AMR after the study period 2 vs 3. | Small sample size, single center, short follow-up |
| C1-INH Berinert | C1r/s; MASPs | 2 | Completed | NCT02936479 (2) | Treatment of AMR | Kidney TX recipients  with acute AMR refractory to standard therapy | 1 | Results were posted, but there was an insufficient sample size to draw conclusions. | No control group (single-arm only), single center, and open-label; the actual enrollment was limited, and co-interventions/confounders were likely due to the defined population. The primary outcome is limited to survival beyond one year. |
| C1-INH Berinert | C1r/s; MASPs | 3 | Terminated due to futility | NCT03221842  (3) | Treatment of refractory AMR | Kidney TX recipients | 63 | Results posted but were not interpretable due to small numbers of subjects and because no subject reached the 48-month follow-up endpoint. | Limited efficacy results due to early termination, potential heterogeneities in the protocol of different centers in administering the standard of care, and a randomized-withdrawal design might affect the findings. |
| C1-INH Berinert | C1r/s; MASPs | 2 | Not yet recruiting | NCT06919003 (4) | Improvement of graft function | Highly HLA-sensitized deceased donor  kidney TX recipients | 180 | No results posted. | The time gap between intervention and outcome evaluation is high, and potential to miss early effects. |
| C1-INH Cinryze | C1r/s; MASPs | 2 | Completed | NCT01147302  (5) | Treatment of AMR | Presensitized kidney TX recipients | 18 | No differences in day-20 pathology or graft survival between the Cinryze and placebo groups; improvement trend in renal function in the Cinryze group. TG incidence was lower in the Cinryze group (0/7 vs 3/7, respectively). | Short follow-up, potential confounding from standard rescue treatments |
| C1-INH Cinryze | C1r/s; MASPs | 3 | Terminated due to futility | NCT02547220 (6) | Treatment of AMR | Kidney TX recipients | 39 | Cinryze group vs placebo: TG at 6 months 50% vs 47.4%. | Early termination and low enrollment; met the pre-specified futility criteria |
| Eculizumab | C5 | 1/2 | Completed | NCT00670774 (7) | Prevention of AMR | Presensitized kidney TX recipients with high levels of DSA | 26 | Incidence of AMR was lower and TX glomerulopathy was less common at year 1 biopsy in the Eculizumab group. | The historical control group, single-arm, open-label |
| Eculizumab | C5 | 2 | Completed | NCT01029587(8) | Prevention of CAPS recurrence after kidney TX | Patients with a history of CAPS after kidney TX | 1 | Not possible to draw conclusions due to insufficient sample size. | The actual enrollment was low, no randomization, no comparator arm |
| Eculizumab | C5 | 1/2 | Terminated due to poor enrolment | NCT01095887 (9) | Prevention of AMR | ABO blood group incompatible living donor kidney TX recipients | 6 | Results were posted, but there was an insufficient sample size to draw conclusions. | The actual enrollment was low, no placebo, or no standard-of-care control arm. |
| Eculizumab | C5 | 1/2 | Terminated due to poor enrolment and funding challenges | NCT01106027 (10) | Prevention of AMR | Positive crossmatch deceased donor  kidney TX recipients | 2 | Results were posted, but there was an insufficient sample size to draw a conclusion. | The actual enrollment was low, no randomization or comparator group |
| Eculizumab | C5 | 1 | Completed | NCT01327573  (11) | Chronic complement-mediated injury | Kidney TX recipients with DSA and worsening kidney function | 16 | Improvement in eGFR change (NS) but no effect on endothelial cell injury. | No survival or rejection outcomes; follow-up is limited to 6 months |
| Eculizumab | C5 | 2 | Terminated with results posted (did not achieve statistical significance for the primary endpoint (post-transplant treatment failure rate at week 9) | NCT01399593 (12) | Prevention of acute AMR in desensitized recipients | Living donor kidney TX recipients | 102 | No new safety concerns. No significant difference compared to the SOC control group, except for the grade 1 AMR subgroup, which showed a lower rate of therapy failure in the Eculizumab group. | Did not achieve statistical significance for the primary endpoint, the follow-up is limited to 9 weeks |
| Eculizumab | C5 | 2 | Completed | NCT01567085 (13) | Prevention of AMR | Presensitized deceased donor kidney TX recipients | 80 | Lower AMR rate in the Eculizumab group compared to the SOC group; no new safety concerns | No randomization or control, potential for heterogeneous, multicenter standard of care |
| Eculizumab | C5 | 2 | Terminated due to lack of efficacy | NCT01895127 (14) | Treatment of AMR | Kidney TX recipients | 11 | No difference between the control and treatment groups. | No randomization, control group, short primary endpoint window, terminated for lack of efficacy; the comparator arm is plasmapheresis + IVIg , a strong standard of care for AMR |
| Eculizumab | C5 | 3 | Completed | NCT02013037  (15) | Prevention of AMR | Heart TX recipients | 36 | Reduced risk of biopsy-proven AMR | Non-randomized, single-center, single-arm, no control groups |
| Eculizumab | C5 | 1/2 | Withdrawn due to the disengagement of the sponsor | NCT02113891 (16) | Treatment of subclinical AMR | Kidney TX recipients with sensitization and  subclinical AMR during the first 3 months post-TX | 0 | No results posted; no patients enrolled. | Historical controls, terminated before starting recruitment, therefore no enrollment |
| Eculizumab | C5 | 1/2 | Not yet recruiting | NCT06453135  (17) | Prevention of AMR | ABO blood group incompatible living donor kidney TX recipients | 30 | No results posted. | No randomized control, single center |
| TNT009  (BIVV09) | C1s | 1 | Completed | NCT02502903  (18) | Treatment for late active AMR | Kidney TX recipients | 10 | No effect on late active AMR but effective blockage of the classical pathway. | Single-center |
| Eculizumab | C5 | 1 | Withdrawn | NCT03468140 (19) | Prevention of post-TX preservation injury | Macrosteatotic liver TX recipients | 0 | No results posted. | Non-randomized, historical controls, low enrollment |
| **Allograft Injury Due to IRI or DGF** | | | | | | | | |  |
| C1-INH Berinert | C1r/s; MASPs | 1/2 | Completed | NCT02134314 (1,20) | Prevention of DGF/IRI | Recipients of deceased donor kidney TX | 70 | No difference in DGF frequency but decreased graft failure in Berinert group. The duration of DGF was shorter in the treated group. | Only deceased-donor kidneys at high DGF risk were included, with exclusions such as any pump-preserved kidneys, which might limit generalizability. |
| C1-INH Berinert | C1r/s; MASPs | 1/2 | Completed | NCT04696146  (21) | Prevention of DGF/​IRI | Deceased  donor kidney TX recipients with allograft considered high-risk for development of DGF (KDPI >80%) | 45 | No difference in DGF incidence was observed, but allograft function was significantly improved in the C1INH-treated group at 6 and 30 months. | The primary outcome regarding the need for dialysis in the first 30 days might be different from the other centers |
| C1-INH Cinryze | C1r/s; MASPs | 1 | Unknown Status | NCT02435732  (22) | Donor pretreatment strategy in recipients of kidney TX with KDPI >60% | Kidney TX recipients with >60% KDPI allograft | 72 | No results posted. | Treating brain-dead donors (with recipient consent dependencies and same-center organ allocation) is logistically tight and may limit generalizability. Randomization occurs in donors, but outcomes are measured in recipients |
| C1-INH Ruconest (rhC1INH) | C1r/s; MASPs | 1 | Unknown Status | NCT03791476  (23) | Prevention of DGF | Deceased donor kidney TX recipients | 20 | No results posted. | Single center  A triple-blind design is advantageous. |
| ARGX-117 | C2 | 2 | Recruiting | NCT05907096  (24) | Prevention of DGF | Deceased donor kidney TX recipients | 20 | No results posted. | Optional enrollment for part B can introduce attrition/selection bias |
| Mirococept | C3b | 1 | Terminated | ISRCTN49958194 (25,26) | Prevention of DGF/IRI | Kidney TX recipients |  | No difference compared to the placebo group. | DGF (dialysis in the first 7 days) might be practice-dependent; pre-emptive recipients are excluded. |
| Eculizumab | C5 | 2 | Terminated pilot study modified to a larger multi-center one (see the CT01919346 study below) | NCT01403389 (27) | Prevention of DGF | Deceased donor kidney TX recipients | 8 | No results posted. | Early termination, limited enrollment, single-center, eligibility criteria might further limit real-world applicability. |
| Eculizumab | C5 | 2 | Completed | NCT01756508 (28) | Prevention and treatment of IRI | Pediatric kidney TX recipients | 57 | Better early graft function and graft morphology, but early graft losses in the Eculizumab group. | No blinding, relatively unvalidated clinical endpoint, included both adult and pediatric population |
| Eculizumab | C5 | 2 | Terminated based on results from the Alexion PROTECT DGF study | NCT01919346 merged with NCT01403389 (27) | Prevention of DGF | Deceased donor kidney TX recipients | 27 | No difference in DGF rate compared to the placebo group. | Small sample size despite being multicenter, terminated early, only first deceased donor kidney transplants were included. |
| Eculizumab | C5 | 2/3 | Completed | NCT02145182 (29) | Prevention of DGF | Kidney TX recipients | 288 | No significant reduction in DGF. | Short-term follow-up |
| Ravulizumab | C5 | 3 | Recruiting | NCT06830798  (30) | Prevention of DGF | Deceased donor kidney TX recipients | 450 | No results posted. | The primary endpoint is time to freedom from dialysis through 90 days. Dialysis-start/stop thresholds may vary by center and clinician unless there is a specific protocol. |
| **Transplantation in Patients with Complement-Mediated Diseases** | | | | | | | | |  |
| Pegcetacoplan  (APL-2) | C3 | 2 | Active, not recruiting | NCT04572854(31) | Post-TX recurrence of  C3G or IC-MPGN | Kidney TX recipients with known C3G or IC-MPGN | 13 | At week 12, pegcetacoplan treatment resulted in significantly reduced glomerular C3 deposition, decreased proteinuria, stable kidney function, and was well tolerated in posttransplant patients with recurrent C3G and IC-MPGN. | The primary endpoint is histological (C3c staining reduction at week 12), open-label design. |
| Iptacopan  (LNP023) | FB | 2 | Completed | NCT03832114  (32) | Assessment of safety and renal function | Patients with C3G (cohort A) and transplanted patients with C3G recurrence (cohort B) | 27 | Significant reductions in urinary protein-to-creatinine ratio and normalization of C3 levels in cohort A and reduction of C3 deposit scores in cohort B, with favorable safety and tolerability. | No blinding or randomization, no placebo or active comparator arm, and short follow-up might limit the encounter of clinically meaningful results. The focus was mainly on pharmacokinetics and biomarkers; the clinical endpoints were limited. |

**Abbreviations:** AE, adverse events; AMR, antibody-mediated rejection; C, complement; CAPS, catastrophic antiphospholipid antibody syndrome; C1-INH, C1 esterase inhibitor; C1r, complement component 1r; C1s, complement component 1s; C3G, C3 glomerulopathy; DGF, delayed graft function; DSA, donor-specific HLA antibodies; eGFR, estimate glomerular filtration rate; ESRD, end-stage renal disease; FB, Factor B; HLA, human leukocyte antigen; IC-MPGN, Immune complex-mediated membranoproliferative glomerulonephritis; IRI, ischemia/reperfusion injury; KDPI, kidney donor profile index; MASPs, mannan-binding lectin-associated serine proteases; NS, not significant; rhC1INH, recombinant human C1-inhibitor; SOC, standard of care; TG, transplant glomerulopathy; TX, transplant.

*The references cited in this table, indicated by consecutive numbers (1–32) following the trial registry identification numbers, are listed in the supplementary material below.

**The assessment of the limitations and quality was limited due to early termination or missing reporting of the trials.

**Supplementary Table 2.** Supplementary References for Table S1.

| **Supplementary References for Table S1** | |
| --- | --- |
| **Reference** | **Full citation** |
| **1** | Jordan SC, Choi J, Aubert O, Haas M, Loupy A, Huang E, Peng A, Kim I, Louie S, Ammerman N *et al*: **A phase I/II, double-blind, placebo-controlled study assessing safety and efficacy of C1 esterase inhibitor for prevention of delayed graft function in deceased donor kidney transplant recipients**. *Am J Transplant* 2018, **18**(12):2955-2964. |
| **2** | **ClinicaTrials.gov NCT02936479: C1-Inhibitor (INH) for Refractory Antibody Mediated Renal Allograft Rejection ClinicalTrials.gov. [(accessed on 04 June 2025)]; Available online:** [**https://clinicaltrials.gov/search?cond=NCT02936479**](https://clinicaltrials.gov/search?cond=NCT02936479) |
| **3** | **ClinicaTrials.gov NCT03221842: Efficacy and Safety of Human Plasma-derived C1-esterase Inhibitor as add-on to Standard of Care for the Treatment of Refractory Antibody Mediated Rejection (AMR) in Adult Renal Transplant Recipients ClinicalTrials.gov. [(accessed on 04 June 2025)]; Available online:** [**https://clinicaltrials.gov/search?cond=NCT03221842**](https://clinicaltrials.gov/search?cond=NCT03221842)**.** |
| **4** | **ClinicaTrials.gov NCT06919003: Improving Deceased-Donor Kidney Transplant Outcomes Via a Single Intragraft Injection of C1 Esterase Inhibitor (IMPROVE TRIAL) (IMPROVE) ClinicalTrials.gov. [(accessed on 04 June 2025)]; Available online:** [**https://clinicaltrials.gov/search?cond=NCT06919003**](https://clinicaltrials.gov/search?cond=NCT06919003)**.** |
| **5** | Montgomery RA, Orandi BJ, Racusen L, Jackson AM, Garonzik-Wang JM, Shah T, Woodle ES, Sommerer C, Fitts D, Rockich K *et al*: **Plasma-Derived C1 Esterase Inhibitor for Acute Antibody-Mediated Rejection Following Kidney Transplantation: Results of a Randomized Double-Blind Placebo-Controlled Pilot Study**. *Am J Transplant* 2016, **16**(12):3468-3478. |
| **6** | Bentall A, Tyan DB, Sequeira F, Everly MJ, Gandhi MJ, Cornell LD, Li H, Henderson NA, Raghavaiah S, Winters JL *et al*: **Antibody-mediated rejection despite inhibition of terminal complement**. *Transpl Int* 2014, **27**(12):1235-1243. |
| **7** | Stegall MD, Diwan T, Raghavaiah S, Cornell LD, Burns J, Dean PG, Cosio FG, Gandhi MJ, Kremers W, Gloor JM: **Terminal complement inhibition decreases antibody-mediated rejection in sensitized renal transplant recipients**. *Am J Transplant* 2011, **11**(11):2405-2413. |
| **8** | **ClinicaTrials.gov NCT01029587: Eculizumab to Enable Renal Transplantation in Patients with History of Catastrophic Antiphospholipid Antibody Syndrome. ClinicalTrials.gov. [(accessed on 04 June 2025)]; Available online:** [**https://clinicaltrials.gov/search?cond=NCT01029587**](https://clinicaltrials.gov/search?cond=NCT01029587)**.** |
| **9** | **ClinicaTrials.gov NCT01095887: Eculizumab to Prevent Antibody-mediated Rejection in ABO Blood Group Incompatible Living Donor Kidney Transplantation (ABOi) ClinicalTrials.gov. [(accessed on 04 June 2025)]; Available online:** [**https://clinicaltrials.gov/search?cond=NCT01095887**](https://clinicaltrials.gov/search?cond=NCT01095887)**.** |
| **10** | **ClinicaTrials.gov NCT01106027: Dosing Regimen of Eculizumab Added to Conventional Treatment in Positive Crossmatch Deceased Donor Kidney Transplant ClinicalTrials.gov. [(accessed on 04 June 2025)]; Available online:** [**https://clinicaltrials.gov/search?cond=NCT01106027**](https://clinicaltrials.gov/search?cond=NCT01106027)**.** |
| **11** | Kulkarni S, Kirkiles-Smith NC, Deng YH, Formica RN, Moeckel G, Broecker V, Bow L, Tomlin R, Pober JS: **Eculizumab Therapy for Chronic Antibody-Mediated Injury in Kidney Transplant Recipients: A Pilot Randomized Controlled Trial**. *Am J Transplant* 2017, **17**(3):682-691. |
| **12** | Marks WH, Mamode N, Montgomery RA, Stegall MD, Ratner LE, Cornell LD, Rowshani AT, Colvin RB, Dain B, Boice JA *et al*: **Safety and efficacy of eculizumab in the prevention of antibody-mediated rejection in living-donor kidney transplant recipients requiring desensitization therapy: A randomized trial**. *Am J Transplant* 2019, **19**(10):2876-2888. |
| **13** | Glotz D, Russ G, Rostaing L, Legendre C, Tufveson G, Chadban S, Grinyo J, Mamode N, Rigotti P, Couzi L *et al*: **Safety and efficacy of eculizumab for the prevention of antibody-mediated rejection after deceased-donor kidney transplantation in patients with preformed donor-specific antibodies**. *Am J Transplant* 2019, **19**(10):2865-2875. |
| **14** | **ClinicaTrials.gov NCT01895127: Efficacy and Safety of Eculizumab for Treatment of Antibody-mediated Rejection Following Renal Transplantation ClinicalTrials.gov. [(accessed on 04 June 2025)]; Available online:** [**https://clinicaltrials.gov/search?cond=NCT01895127**](https://clinicaltrials.gov/search?cond=NCT01895127) |
| **15** | Patel JK, Coutance G, Loupy A, Dilibero D, Hamilton M, Kittleson M, Kransdorf E, Azarbal B, Seguchi O, Zhang X *et al*: **Complement inhibition for prevention of antibody-mediated rejection in immunologically high-risk heart allograft recipients**. *Am J Transplant* 2021, **21**(7):2479-2488. |
| **16** | **ClinicaTrials.gov NCT02113891: Eculizumab Therapy for Subclinical Antibody-mediated Rejection in Kidney Transplantation (TAMARCIN) ClinicalTrials.gov. [(accessed on 04 June 2025)]; Available online:** [**https://clinicaltrials.gov/search?cond=NCT02113891**](https://clinicaltrials.gov/search?cond=NCT02113891)**.** |
| **17** | **ClinicaTrials.gov NCT06453135: Eculizumab for Prevention of Antibody-Mediated Rejection in ABO-Incompatible Living Donor Kidney Transplantation. ClinicalTrials.gov. [(accessed on 04 June 2025)]; Available online:** [**https://clinicaltrials.gov/search?cond=NCT06919003**](https://clinicaltrials.gov/search?cond=NCT06919003)**.** |
| **18** | Jager U, D'Sa S, Schorgenhofer C, Bartko J, Derhaschnig U, Sillaber C, Jilma-Stohlawetz P, Fillitz M, Schenk T, Patou G *et al*: **Inhibition of complement C1s improves severe hemolytic anemia in cold agglutinin disease: a first-in-human trial**. *Blood* 2019, **133**(9):893-901. |
| **19** | **ClinicaTrials.gov NCT03468140: Pilot Trial of Eculizumab Therapy to Reduce Preservation Injury in Human Macrosteatotic Liver Transplantation ClinicalTrials.gov. [(accessed on 04 June 2025)]; Available online:** [**https://clinicaltrials.gov/search?cond=NCT03468140**](https://clinicaltrials.gov/search?cond=NCT03468140) |
| **20** | Huang E, Vo A, Choi J, Ammerman N, Lim K, Sethi S, Kim I, Kumar S, Najjar R, Peng A *et al*: **Three-Year Outcomes of a Randomized, Double-Blind, Placebo-Controlled Study Assessing Safety and Efficacy of C1 Esterase Inhibitor for Prevention of Delayed Graft Function in Deceased Donor Kidney Transplant Recipients**. *Clin J Am Soc Nephrol* 2020, **15**(1):109-116. |
| **21** | Huang E, Ammerman N, Vo A, Hou J, Kumar S, Badash N, Falk B, Hernando K, Gillespie M, Kim IK *et al*: **Back-table intra-arterial administration of C1 esterase inhibitor to deceased donor kidney allografts improves posttransplant allograft function: Results of a randomized double-blind placebo-controlled clinical trial**. *Am J Transplant* 2025. |
| **22** | **ClinicaTrials.gov NCT02435732: CINRYZE as a Donor Pre-treatment Strategy in Kidney Recipients of KDPI>60%. ClinicalTrials.gov. [(accessed on 04 June 2025)]; Available online:** [**https://clinicaltrials.gov/search?cond=NCT02435732**](https://clinicaltrials.gov/search?cond=NCT02435732)**.** |
| **23** | **ClinicaTrials.gov NCT03791476: RUCONEST® as a Therapeutic Strategy to Reduce the Incidence of Delayed Graft Function. ClinicalTrials.gov. [(accessed on 04 June 2025)]; Available online:** [**https://clinicaltrials.gov/search?cond=NCT03791476**](https://clinicaltrials.gov/search?cond=NCT03791476)**.** |
| **24** | **ClinicaTrials.gov NCT05907096: ARGX-117 in Deceased Donor Kidney Transplant Recipients at Risk for Delayed Graft Function (VARVARA). ClinicalTrials.gov. [(accessed on 04 June 2025)]; Available online:** [**https://clinicaltrials.gov/search?cond=NCT05907096**](https://clinicaltrials.gov/search?cond=NCT05907096)**.** . |
| **25** | Kassimatis T, Greenlaw R, Hunter JP, Douiri A, Flach C, Rebollo-Mesa I, Nichols LL, Qasem A, Danzi G, Olsburgh J *et al*: **Ex vivo delivery of Mirococept: A dose-finding study in pig kidney after showing a low dose is insufficient to reduce delayed graft function in human kidney**. *Am J Transplant* 2021, **21**(3):1012-1026. |
| **26** | Kassimatis T, Qasem A, Douiri A, Ryan EG, Rebollo-Mesa I, Nichols LL, Greenlaw R, Olsburgh J, Smith RA, Sacks SH *et al*: **A double-blind randomised controlled investigation into the efficacy of Mirococept (APT070) for preventing ischaemia reperfusion injury in the kidney allograft (EMPIRIKAL): study protocol for a randomised controlled trial**. *Trials* 2017, **18**(1):255. |
| **27** | Schroppel B, Akalin E, Baweja M, Bloom RD, Florman S, Goldstein M, Haydel B, Hricik DE, Kulkarni S, Levine M *et al*: **Peritransplant eculizumab does not prevent delayed graft function in deceased donor kidney transplant recipients: Results of two randomized controlled pilot trials**. *Am J Transplant* 2020, **20**(2):564-572. |
| **28** | Kaabak M, Babenko N, Shapiro R, Zokoyev A, Dymova O, Kim E: **A prospective randomized, controlled trial of eculizumab to prevent ischemia-reperfusion injury in pediatric kidney transplantation**. *Pediatr Transplant* 2018, **22**(2). |
| **29** | **ClinicaTrials.gov NCT02145182: Prevention of Delayed Graft Function Using Eculizumab Therapy (PROTECT Study). ClinicalTrials.gov. [(accessed on 04 June 2025)]; Available online:** [**https://clinicaltrials.gov/ct2/show/NCT02145182**](https://clinicaltrials.gov/ct2/show/NCT02145182) |
| **30** | **ClinicaTrials.gov NCT06830798: Double-blind, Randomized, Placebo-controlled, Multicenter Study to Evaluate the Efficacy and Safety of Ravulizumab Administered Intravenously in Adult Participants at High Risk of Delayed Graft Function After Kidney Transplantation (AWAKE). ClinicalTrials.gov. [(accessed on 04 June 2025)]; Available online:** [**https://clinicaltrials.gov/search?cond=NCT06830798**](https://clinicaltrials.gov/search?cond=NCT06830798)**.** |
| **31** | Bomback AS, Daina E, Remuzzi G, Kanellis J, Kavanagh D, Pickering MC, Sunder-Plassmann G, Walker PD, Wang Z, Ahmad Z *et al*: **Efficacy and Safety of Pegcetacoplan in Kidney Transplant Recipients With Recurrent Complement 3 Glomerulopathy or Primary Immune Complex Membranoproliferative Glomerulonephritis**. *Kidney Int Rep* 2025, **10**(1):87-98. |
| **32** | Wong E, Nester C, Cavero T, Karras A, Le Quintrec M, Lightstone L, Eisenberger U, Soler MJ, Kavanagh D, Daina E *et al*: **Efficacy and Safety of Iptacopan in Patients With C3 Glomerulopathy**. *Kidney Int Rep* 2023, **8**(12):2754-2764. |
